# Supplementary material for: Imaging methods are vastly underreported in biomedical research
Source: eLife. 2020 Aug 11;9:e55133. doi: 10.7554/eLife.55133 (PMC7434332; doi:10.7554/eLife.55133)
Supplement: Supplementary file 2. [file elife-55133-supp2.docx]

**Appendix 2. Preliminary checklist for image acquisition reporting**

Wide field fluorescence

- Stand model and brand
- Acquisition software and version
- Objective correction, magnification, and numerical aperture
- Camera brand and model
- Camera binning and EM gain (if applicable)
- Illuminator type, model, and brand
- Effective excitation band for each dye
- Effective emission band for each dye
- Exposure time
- Voxel size
- Frame/volume interval (time-lapse only)

Fluorescence laser scanning confocal

- Stand model and brand
- Acquisition software
- Objective correction, magnification, and numerical aperture
- Confocal scan head model and brand
- Laser light sources
- Excitation wavelengths for each dye
- Effective emission band for each dye
- Sequential or simultaneous acquisition
- Pixel dwell time
- Image size in pixels
- Laser zoom
- Confocal aperture
- Voxel size
- Frame/volume interval (time-lapse only)

Figure assignment

In the case the manuscript contains images acquired with different instruments or imaging modalities, or the same instrument and modality but with significantly different experimental conditions (for example, different objective) those should be listed, and the figures in the paper ascribed to the Materials and Methods experimental setup used (see example in doi.org/10.1083/jcb.201802102).
